# Differential expression of the adult specifier E93 in the strepsipteran *Xenos vesparum* Rossi suggests a role in female neoteny

**DOI:** 10.1038/s41598-018-32611-y

**Published:** 2018-09-21

**Authors:** S. Chafino, D. López-Escardó, G. Benelli, H. Kovac, E. Casacuberta, X. Franch-Marro, J. Kathirithamby, D. Martín

**Affiliations:** 1 Institute of Evolutionary Biology (CSIC-Universitat Pompeu Fabra) Passeig Marítim de la Barceloneta 37-49, 08003 Barcelona, Spain; 2 Department of Zoology, University of Oxford, Oxford, United Kingdom; 3 Department of Agriculture, Food and Environment, University of Pisa via del Borghetto 80, 56124 Pisa, Italy; 4 Institut für Biologie, Universitaet Graz, Universitaetsplatz 2, A-8010 Graz, Austria

## Abstract

Holometaboly is a key evolutionary innovation that has facilitated the spectacular radiation of insects. Despite the undeniable advantage of complete metamorphosis, the female of some holometabolous species have lost the typical holometabolous development through neoteny. In *Xenos vesparum* Rossi (Strepsiptera: Stylopidae), a derived species of the holometabolous endoparasitic order Strepsiptera, neotenic females reach sexual maturity without the pupal and the imaginal stages, thus retaining their larval morphology (with the exception of the anterior part of the body or cephalothorax), while males undergo normal pupal-based metamorphosis. Expression of the “adult-specifier” E93 factor has been shown to be required for proper metamorphosis in holometabolous insects. Here, we investigated the involvement of E93 in female neoteny by cloning *XvE93*. Interestingly, while we detected a clear up-regulation of *XvE93* expression in pupal and adult stages of males, persistent low levels of *XvE93* were detected in *X*. *vesparum* females. However, a specific up-regulation of *XvE93* was observed in the cephalothorax of late 4^th^ female instar larva, which correlates with the occurrence of neotenic-specific features in the anterior part of the female body. Moreover, the same expression dynamic in the cephalothorax and abdomen was also observed for other two critical metamorphic regulators, the anti-metamorphic *XvKr-h1* and the pupal specifier *XvBr-C*. The specific up-regulation of *XvE93* and *XvBr-C* in the female cephalothorax seems to be the result of an increase in 20-hydroxyecdysone (20E) signaling in this region for we detected higher expression levels of the 20E-dependent nuclear receptors *XvHR3* and *XvE7*5 in the cephalothorax. Overall, our results detect a sex-specific expression pattern of critical metamorphic genes in *X*. *vesparum*, suggesting that neoteny in Strepsiptera results from the modification of the normal expression of *E93*, *Br-C* and *Kr-h1* genes.

## Introduction

From their origin approximately 400 Mya^1,2^, insects have developed three basic types of metamorphic development: (i) ametaboly (Apterygota), the most primitive type that is characterized by the absence of a morphological transformation between the wingless immature individuals and the adults; (ii) hemimetaboly (Exopterygota), in which the juvenile wingless nymphs resemble miniature adults and metamorphose into winged adults during the last juvenile instar; and (iii) holometaboly (Endopterygota), in which the crawling juvenile larvae undergo a dramatic morphological transformation to form the winged adult through a two-stage metamorphic process bridged by the holometabolous-specific intermediate pupal stage^3,4^. Taking into consideration that holometaboly has facilitated the most spectacular radiation of animals, complete metamorphosis can be considered as one of the key evolutionary innovation in insect evolution^5,6^. And yet, in spite of the clear evolutionary advantages of holometaboly, the female of a number of holometabolan species have lost the typical complete metamorphic transformation.

The loss of complete metamorphosis in holometabolous insects is exclusively found in females and results from the disappearance of pupal and post-metamorphic stages, a process named paedomorphosis. One particular strategy leading to paedomorphosis is neoteny, where the larval morphology is retained and sexual maturity is attained without a pupal or an imaginal stage^7,8^. Interestingly, whereas holometaboly originated only once in insect evolution from ancestors exhibiting hemimetabolous development^2^, neoteny in holometabolous insects has occurred independently several occasions in the beetle superfamily Elateroidea^9,10^ and once in derived lineages of the endoparasitic order Strepsiptera^11–13^. Despite the interest in how insect neoteny has evolved, the molecular mechanisms underlying the loss of complete metamorphosis in holometabolous neotenic females, however, remain poorly understood.

**Figure 1:**
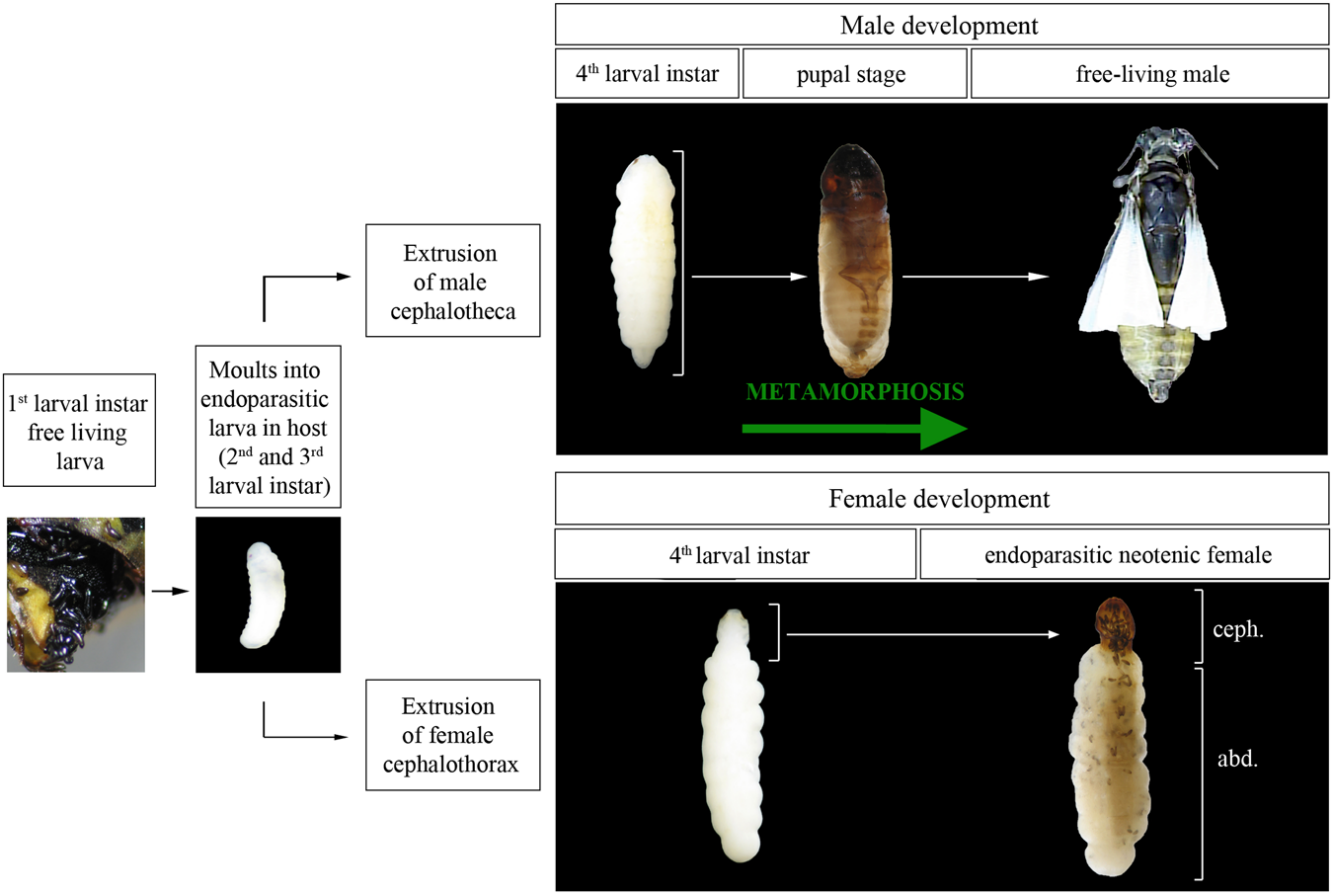
Life cycle of *Xenos vesparum*. Free living 1^st^ instar planidium enters a wasp nests to parasitize host larva. Upon entering the host, the planidium molts to an apodous endoparasitic 2^nd^ instar larva, which successively molts two additional times. The male 4^th^ instar larva undergoes metamorphosis through pupation (green arrow), and after, it extrudes through the abdomen of the wasp cuticle. The apolised cuticles of the larval instars form the puparium. The free-living male emerges from the puparium as a winged adult. In contrast, the 4^th^ instar female larva does not undergo the pupal-based metamorphic transformation but develops a cephalothorax, which is extruded through the host. The cephalothorax contains adult-specific structures that facilitate the neotenic female in insemination and the release of the planidium, and also contains glands that release pheromones to attract the male. The abdomen (abd.) and the cephalothorax (ceph.) regions in the neotenic female are marked.

Strepsiptera are a small, bizarre holometabolous order of obligate entomophagous endoparasitoids with extreme sexual dimorphism^11–15^. Males and females of the early branching order of Strepsiptera, the Mengenillidae, emerge to pupate externally from the host. In contrast, males of the families of the derived suborder Stylopidia pupate endoparasitically in the host and emerge as free-living adults, while females remain obligate endoparasites in the host and are neotenic. The neotenic female Stylopidia, such as *Xenos vesparum* Rossi, produces 1^st^ instar larvae (planidia) which have rudimentary eyes, thoracic limbs, sclerotized cuticle, and are dedicated to host-seeking. Upon finding and entering a host, the planidium molts to a soft cuticle-apodous endoparasitic 2^on^ instar larva. After three consecutive endoparasitic stages where no ecdysis occurs, the male larva extrudes the head (cephalotheca) through the host cuticle and continues the typical holometabolous development whereby it undergoes complete metamorphosis through pupation. At the end of pupation, the male emerges from the endoparasitic puparium as a free-living winged adult. In contrast, the female after the fourth larval instar extrudes the head, thoracic and first abdominal regions (cephalothorax) though the host cuticle and remains endoparasitic in the mobile host. A few days (3–4 days) after extrusion of the cephalothorax, the neotenic female assumes a calling position^16^. During this calling, the female produces a potent pheromone that attracts the male^17–19^. The male then inseminates the female through the brood canal opening in the cephalothorax^16^. After viviparous development in the endoparasitic female, the motile 1^st^ instar planidia emerges through the brood canal opening in the cephalothorax^11,13,15^. Neotenic female Strepsiptera resembles a “bag of eggs” with an extruded cephalothorax without any external adult characteristics, such as antennae, legs and wings, and whose primary function is to be a repository for eggs^13,19,20^ (Fig. 1). Therefore, it could be considered that total endoparasitism in Stylopidia females has led to the simplification of their body plan and represents a state of extreme neoteny. However, it is worth noting that although the abdomen remains larviform, particular structures to aid mating, release of the planidia larvae and the pheromone glands are specifically found in the cephalothorax of neotenic females^20^ (Fig. 1).

From an endocrine perspective, the metamorphic transition in holometabolous insects is controlled by the sesquiterpenoid juvenile hormone (JH) produced by the *corpora allata* glands (CA)^21–25^. The presence of JH during pre-ultimate larval stages prevents premature metamorphosis though the transcriptional induction of the anti-metamorphic transcription factor-encoding gene *Krüppel-homolog 1* (*Kr-h1*)^26,27^. Conversely, the disappearance of JH and the down-regulation of *Kr-h1* at the onset of the last larval instar trigger the metamorphic transformations to the pupal stage by allowing the up-regulation of the transcription factor *Broad-complex* (*Br-C*) by the steroid hormone 20-hydroxyecdysone (20E)^28,29^. During the final part of the last larval instar, Br-C controls the correct switch between larval and pupal forms, thus acting as the “pupal specifier”^28–32^. In pre-ultimate larval stages, Kr-h1 binds to the promoter region of *Br-C* and inhibits its expression, thus preventing larvae to undergo precocious larval-pupal transformation^33^. Finally, after pupation, a third critical transcription factor, E93, is strongly up-regulated by 20E and controls the transition from the pupa to the adult, thus acting as the “adult specifier”^34^. During the pupal stage, E93 also represses the expression of *Kr-h1* and *Br-C*, thus ensuring the elimination of the two factors whose presence during this period is detrimental for proper adult differentiation^1,34^. Similar to the repressive activity on *Br-C*, Kr-h1 also binds to the promoter region of *E93*, suppressing its expression before the pupal stage and preventing larvae to undergo precocious larval-adult metamorphosis^35^. Due to their critical importance in the control of metamorphosis in holometabolous and hemimetabolous insects, Kr-h1, Br-C and E93 form what we have recently defined as the *Metamorphic Gene Network* (MGN)^1^.

In order to characterize the genetic control of neoteny in holometabolous insects, a recent pilot study proposed that the appearance of neotenic females in Strepsiptera is linked to the modification of regulatory pathways that underlie pupal determination^36^. The authors identified XvBr-C in *X*. *vesparum* and found upregulation in males at the larval-pupal transition but not in females. This work suggested that lack of *XvBr-C* upregulation might be one of the reasons underlying the loss of complete metamorphosis in neotenic females. However, it has been shown in *T*. *castaneum* that depletion of *TcBr-C* does not result in the maintenance of larval status but rather to a developmental arrest of the animal at the larval-pupal transition with knockdown animals showing a mix of larval, pupal and adult features^1,28–31^, thus suggesting that additional factors are required to induce complete metamorphosis.

Here, we further investigate the postulated link between the alteration of the pupal determination program controlled by the MGN and the occurrence of adult neotenic *X*. *vesparum* females. Due to the critical role of E93 in the formation of the adult form, we have identified *X*. *vesparum* E93 homolog (XvE93). Then, we have compared the expression dynamics of *XvE93* in males and females during the metamorphic transition. Because the female is composed of a posterior abdomen that is larval-like and an anterior cephalothorax that has features for insemination and release of the 1^st^ instars and the pheromone glands we analyzed the differential expression of *XvE93* in these two regions of the female. Moreover, to complete the identification of the factors of the MGN, we have isolated a fragment of the anti-metamorphic Kr-h1 (XvKr-h1) factor and have measured its expression levels during the larva-to-adult transition in *X*. *vesparum* males and females. Finally, to complete the analysis of the MGN in the regulation of neoteny, we have measured the expression levels of *XvBr-C* in the cephalothorax and the abdomen of X. vesparum females. Our results strongly suggest that the low levels of *XvE93* expression in *X*. *vesparum* females underlie the evolution of neotenic development.

## Material and Methods

### Strepsiptera collection

*X*. *vesparum* specimens used for cloning and gene expression analysis were collected from the following locations:

(*i*) *X*. *vesparum* parasitic in *Polistes dominulus* were collected in Italy in 2017, in Tuscany (Central Italy) Via Madonna del Piano, 6. Sesto Fiorentino, It-50019, Italy; N43°49.064′ E11°12.263′ by Helmut Kovac; (*ii*) *X*. *vesparum* parasitic in *P*. *dominulus* were collected in 2014, Tuscany (Central Italy) from University of Pisa farm, San Piero a Grado, Pisa (43°40′01.6″N 10°20′21.0″E) and in a private winery, “La Sughera” farm, Loc. Tonini, Spianate, Altopascio, Lucca (43°48′00.2″N 10°42′02.6″E) by Giovanni Benelli and J. Kathirithamby; (*iii*) *X*. *vesparum* parasitic in *P*. *dominulus* were collected in 2017 in Austria, Gschwendterstraße 76, A-8062 Kumberg, Austria (N47 10.721 E15 34.373) by H. Kovac; (*iv*) *X*. *vesparum* parasitic in *P*. *dominulus* were collected from nests in plastic bottles in 2016 in Fauglia, Pisa, by Consolato Latella and J. Kathirithamby. In all cases, specimens were dissected immediately in saline and fast frozen at −80 °C, or fast frozen and dissected for identification of the endoparasitic stages.

### *X. vesparum* transcriptome assembly for sequence analysis

Raw reads were downloaded from NCBI SRA repository with accession numbers SRR1784898 and SRR1784897 which belong to RNA-seq studies on a *X*. *vesparum* female 4^th^ instar larvae and neotenic females, respectively. Two independent transcriptomes assemblies were carried out with rnaSPAdes^37^ v.0.0.1. Before the assembly, raw reads were trimmed with Trimmomatic^38^ v3.059 using the following options ILLUMINACLIP:/adapters/TruSeq. 3-PE.fa:2:30:10 SLIDINGWINDOW:4:28 MINLEN:50 in order to remove Truseq adapters and low quality reads. Next, by using known E93, Kr-h1, HR3 and E75 protein sequences from *Blattella germanica* and *Drosophila melanogaster*, we performed tBLASTn^39^ searches (BLAST v.2.2.31) in order to identify the putative transcripts in *X*. *vesparum* and design primers for further experimental confirmation of gene sequences.

### Phylogenetic analysis of E93

To understand the phylogenetic relationship of E93 proteins, amino acid sequences from E93 proteins were collected from different insect taxa, including that of *X*. *vesparum* as well as from two arachnid species as an outgroup (Supplementary Table 1), and aligned using MAFFT^40^ v7.299b L-INS-I with 1000 iterations. Ambiguously aligned positions were trimmed using trimAl^41^ v14, with the automated 1 algorithm. The best substitution model for phylogenetic inference was selected using IQ-TREE^42^ with the TESTNEW model selection procedure and following the BIC criterion. The LG substitution matrix with a 4-categories discrete Γ distribution and allowing for invariant sites was selected as the best-fitting model. Maximum likelihood inferences were performed with IQ-TREE, and statistical supports were drawn from 1,000 ultrafast bootstrap values with a 0.99 minimum correlation as convergence criterion^43^, and 1,000 replicates of the SHlike approximate likelihood ratio test^44^.

### Pipsqueak Domain Similarity Analysis

A semi-automated BLAST and HMM-based search was conducted with the online Pfam database^45^ and the Pfam HMM profile for pipsqueak domain. *E93* sequences of *D*. *melanogaster (NP_6*5*2002*.*2)*, *Tribolium castaneum (KYB25179*.*1)*, *B*. *germanica (CCM97102*.*1)*, *Bombyx mori (AIL29268*.*1)*, *Apis mellifera (BAB64310*.*1)* and *Zootermopsis nevadensis (KDR22086*.*1)*, *Caenorhabditis elegans* (*AB236333*.*1*) and *Homo sapiens* (*AAH53359*.*1*) were obtained from GenBank database, were used for the analysis.

### Quantitative real-time reverse transcriptase polymerase chain reaction (qRT-PCR)

Total RNA from individual *X*. *vesparum* specimens was extracted using the GenEluteTM Mammalian Total RNA kit (Sigma). cDNA synthesis was carried out as previously described^46,47^. Relative transcript levels were determined by quantitative real-time PCR (qPCR), using Power SYBR Green PCR Mastermix (Applied Biosystems). To standardize the quantitative real-time RT-PCR (qPCR) inputs, a master mix that contained Power SYBR Green PCR Mastermix and forward and reverse primers was prepared to a final concentration of 100 µM for each primer. The qPCR experiments were conducted with the same quantity of tissue equivalent input for all treatments, and each sample was run in duplicate using 2 µl of cDNA per reaction. As a reference, same cDNAs were subjected to qRT-PCR with a primer pair specific for *X*. *vesparum* Ribosomal 18 S^36^. All the samples were analyzed on the iCycler iQReal Time PCR Detection System (Bio-Rad). Primer sequences used for qPCR for *X vesparum* are:

XvE93-F: *5*′-GGTACAACGCGGTGAAATGTC-3′XvE93-R: 5′-GTTTTCGTGGCCGCATTAAATGC-3′XvKrh1-F: 5′-TATGCGACGATGTACGCTTA-3′XvKrh1-R: 5′-CTTGCACGTTTAACACGTCAT-3′XvHR3-F: 5′-CTACGAGCAAACACCATCGA-3′XvHR3-R: 5′-GGATTGTAATAAGTCGTATACG-3′XvE75-F: 5′-GAAAGAGGAACCAACAAGTTC-3′XvE75-R: 5′-CTTACAACCTTCACATGAATGA-3′XvBr-C-F: 5′-GCAGCATTACCTCTGCTT-3′XvBr-C-R: 5′-CGAAAATATGGGCTGCAG-3′Xv18S-F: 5′-TGCGGCGTATCTTTCAATTGT*-3*′Xv18S-R: *5*′*-*CTGCCTTCCTTAGATGTGGT-3′.

## Results and Discussion

### Identification and phylogenetic analysis of XvE93

**Figure 2:**
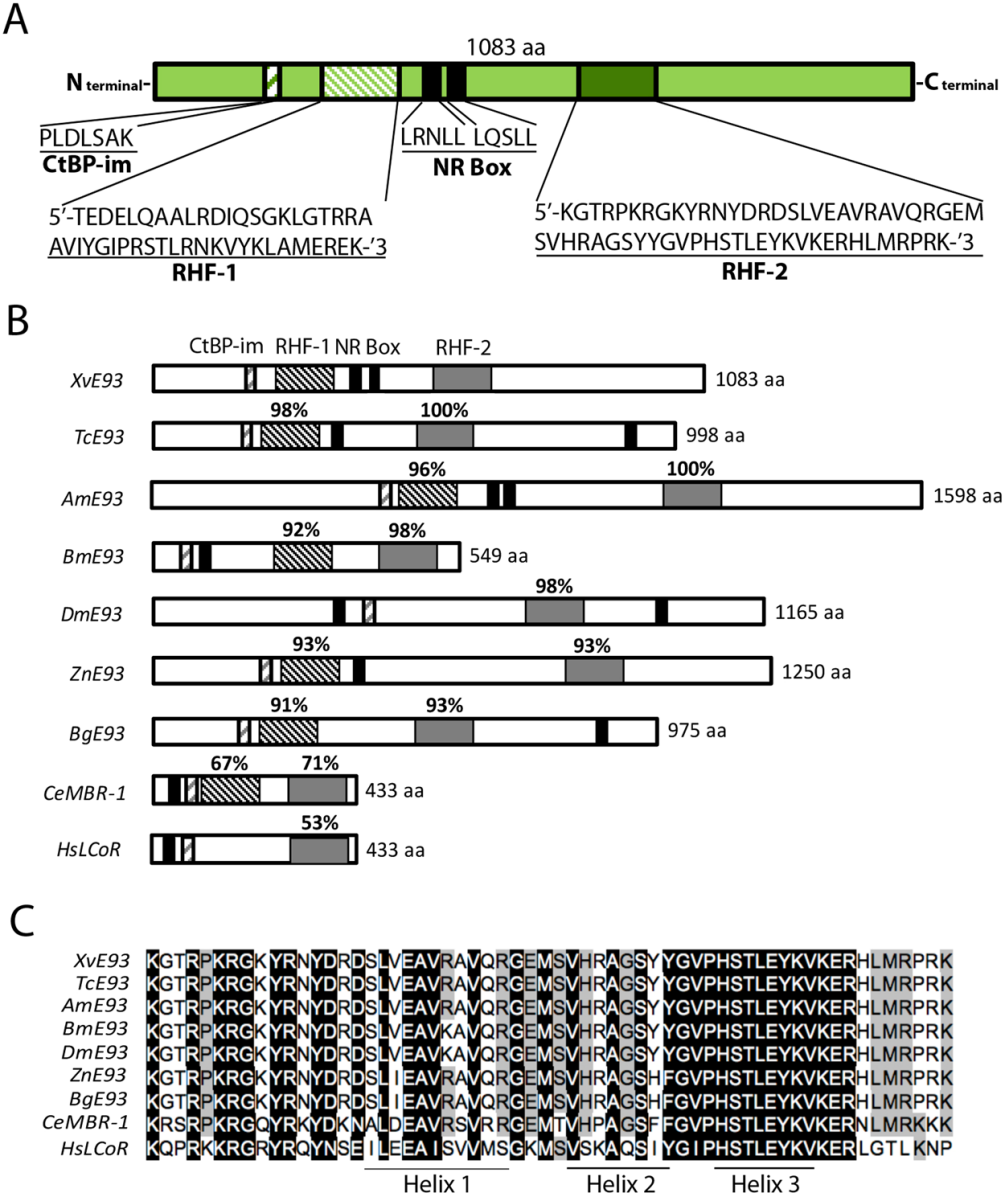
The structure of *Xenos vesparum* XvE93. (**A**) Predicted amino acid sequence of XvE93. The two HTH-DNA binding motifs, RHF1 (grey box) and RHF2 (stripped box), are indicated. The two NR-box are boxed in black. The CtBP-interaction motif (CtBP-im) is single-line stripped boxed. (**B**) Comparison of the domain structure of E93 homologs. *Tribolium castaneum* (TmE93), *Apis mellifera* (AmE93), *Bombyx mori* (BmE93), *Drosophila melanogaster* (DmE93), *Zootermopsis nevadensis* (ZnE93), *Blattella germanica* (BgE93), *Caenorhabditis elegans* (CeE93) and *Homo sapiens* (HsLCoR). The percentages indicate the sequence identities within each domain. (**C**) Comparison of the RHF2 domain of XvE93 with those from other E93 homologs. Amino acid residues highly conserved in E93 insects, MBR-1 from *C*. *elegans* and human LCoR are indicated by a reverse background, whereas moderate conserved residues are shaded in grey. The positions of the three helices are indicated by horizontal bars.

*X*. *vesparum E93* (XvE93) full-length sequence was obtained by the assembly of *X*. *vesparum* transcriptomes from 4^th^ instar female larvae and neotenic females (GenBank accession number MH220841). A single cDNA that corresponds to a transcription factor of 1083 amino acid was identified (Fig. 2A and Supplementary Fig. 1). The comparison with other E93 sequences (Fig. 2B,C), revealed that XvE93 possess two helix-turn-helix (HTH) DNA binding motifs of the pipsqueak family, termed RHF1 and RHF2 (Fig. 2A,B). RHF-1 presents 91–98% similarity compared to other insects and a 67% compared to the *Caenorhabditis elegans* homolog MBR-1, while RHF-2 presents 93–100% similarity compared to other insects, 71% compared to MBR-1 and 53% compared to the human homolog Ligand Co-Repressor (LCoR). Although *D*. *melanogaster* DmE93 and human LCoR have only the RHF-2 domain, the two RHF DNA-binding motifs have been shown to be critical for proper induction of 20E dependent genes in the lepidepteran *B*. *mori*^48^, suggesting that both domains, RHF-1 and RHF-2, are required for binding to DNA.

An interesting feature of all E93 homologs is the presence of at least one nuclear receptor interaction motif (NR-box; also named as LXXLL motif). Interestingly, XvE93 contains two of such motifs (Fig. 2A,B). Through these motifs LCoR interacts with different hormone-bound nuclear receptors attenuating their transcriptional activity^49,50^. Likewise, *B*. *mori* BmE93 interacts through the NR-box with ultraspiracle (USP), a nuclear receptor that dimerizes with the ecdysone receptor (EcR) to act as the functional 20E receptor, to impair the transcriptional activity of the heterodimer^48^. Finally, a third conserved domain present in XvE93 is the co-repressor C-terminal-binding protein interaction motif (CtBP-im) (Fig. 2A,B). Overall, the highly conserved protein structure observed in all E93 homologs, including that of *X*. *vesparum*, suggests that XvE93 functions through similar molecular mechanisms.

**Figure 3:**
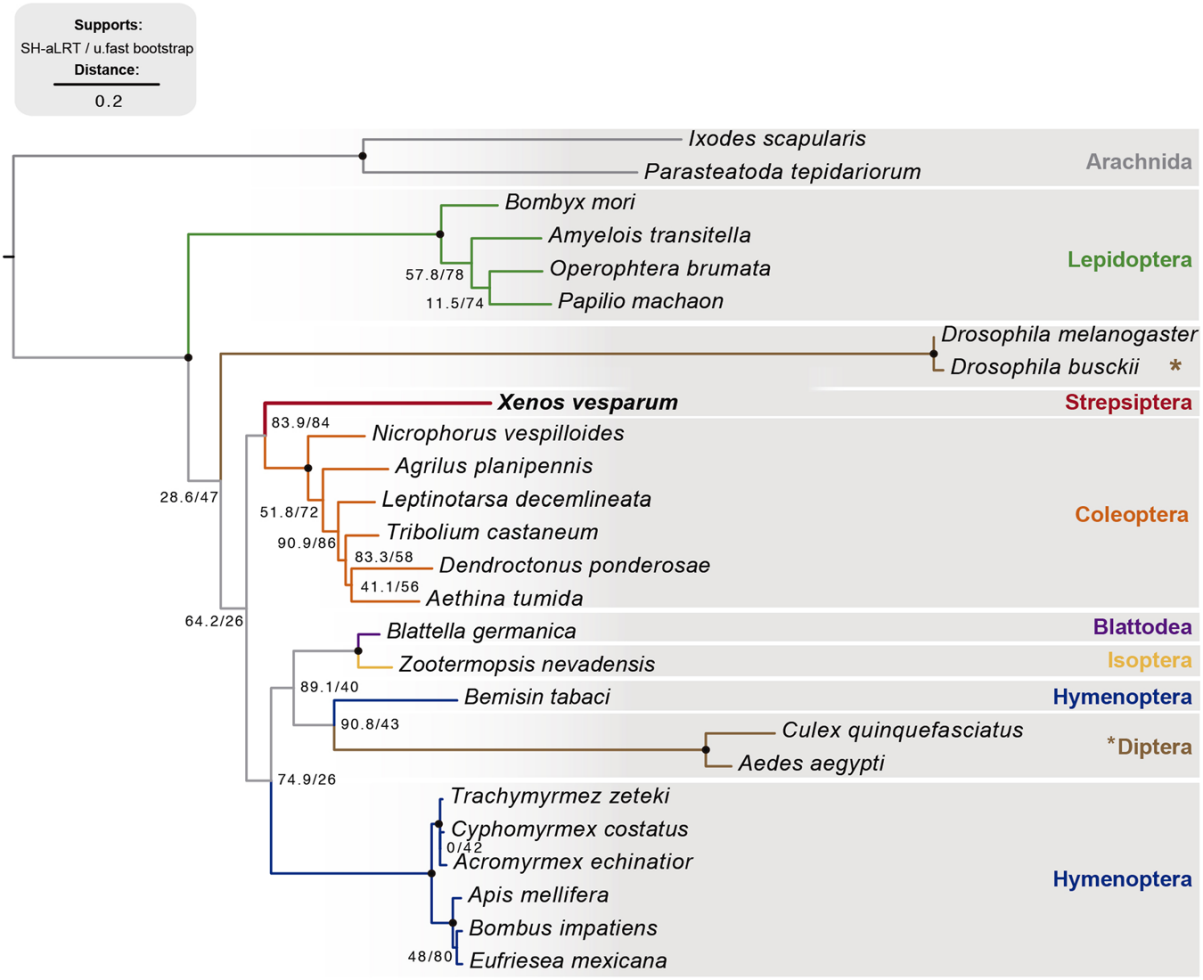
Phylogenetic analysis of E93 proteins. Phylogenetic tree based in protein sequences of E93 from 24 different insect taxa including the E93 sequence of *Xenos vesparum* described in this study. Two Arachnida E93 sequences are used as outgroup. Branch colors at the tip of the tree indicated the order of the different species used in this analysis. *X*. *vesparum* is highlighted in bold.

On the other hand, maximum-likelihood analysis of E93 sequences, using the sequences of arachnids E93 as outgroup, showed that XvE93 sequence grouped with those of coleopteran species, which is consistent with phylogenomic inferences^2,51^ (Fig. 3). A remarkable feature of the tree was the different length of the branches. Diptera, and specially flies, had the longest lengths, clearly indicating a faster rate of divergence of these sequences with respect to other insect sequences, whereas non-dipteran branches were shorter, indicating a higher conservation of these sequences.

### Differential expression of *XvE93* in males and females of *X*. *vesparum*

**Figure 4:**
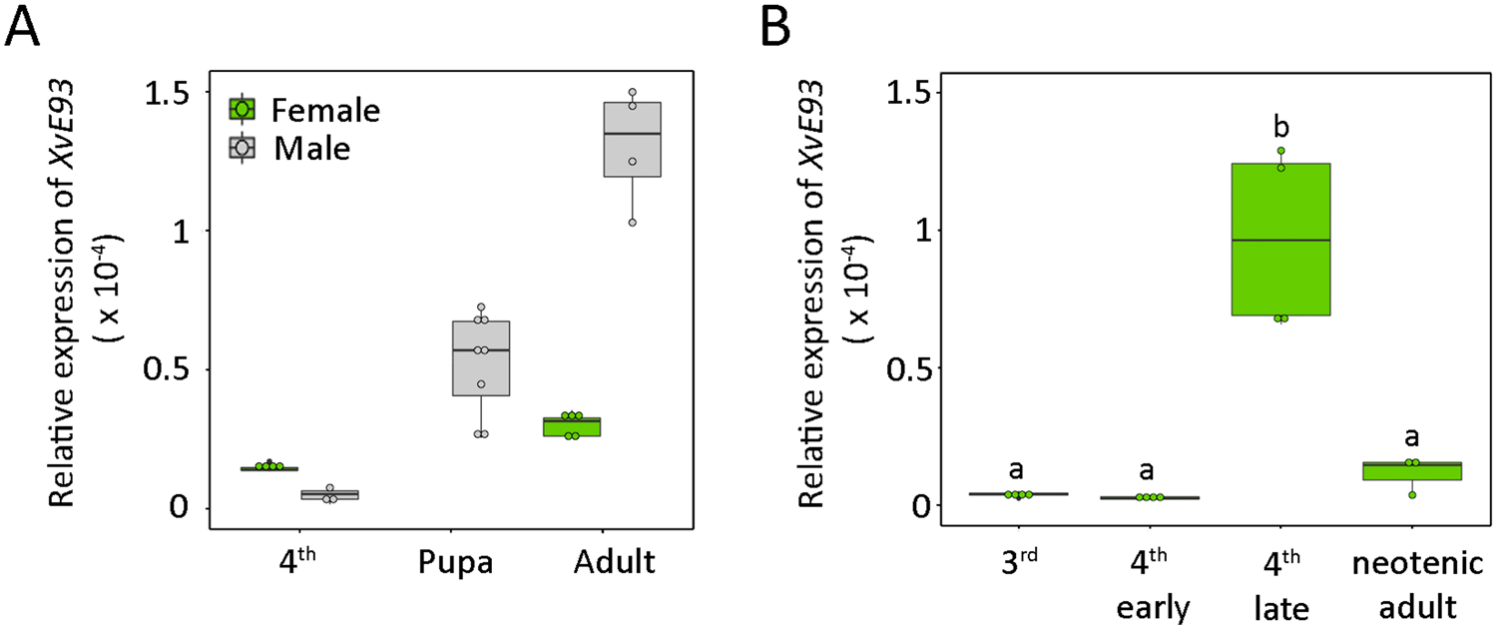
Developmental expression profiles of *Xenos vesparum XvE93* in the transition from larvae to adults. (**A**) *XvE93* mRNA levels were measured by qRT-PCR in the larva-to-adult transition in male (4^th^, pupa, adult) and female (4^th^ and adult) whole bodies. (**B**) *XvE93* mRNA levels (qRT-PCR) in the whole body of 3^th^, early and late 4^th^ and neotenic females. Transcript abundance values in panels A and B are normalized against the *XvRibosomal 18S* transcript. The range of expression is shown by Boxplot: boxes demarcate the upper and lower quartiles, while the heavy bar indicates the median value of normalized expression. Whiskers extend to the most extreme values. Points represent each individual measurement. Different letters in panel B represent groups with significant differences according to ANOVA test (Tukey, p ≤ 0.005).

As a first step towards the characterization of *E93* in neoteny, we measured the expression levels of *XvE93* in the larva-to-adult transition of *X*. *vesparum*. Males of *X*. *vesparum* progress through four larval stages (one free-living planidium stage and three endoparasitic stages) before undergoing endoparasitic pupation, when the metamorphic transition results in the formation of free-living winged adults (Fig. 1). In contrast, *X*. *vesparum* females pass through four larval molts (one free-living planidium stage and three endoparasitic stages) which then transform directly into endoparasitic larviform neotenic animals without transiting through the metamorphic-pupal stage (Fig. 1). As shown in Fig. 4A, mRNA expression levels of *XvE93* in males were low in 4^th^ larval instar but significantly increased in the pupal and adult stages. In contrast, *XvE93* levels in females were very low in 4^th^ instar larvae and did not show any detectable increase in neotenic adults (Fig. 4A).

**Figure 5:**
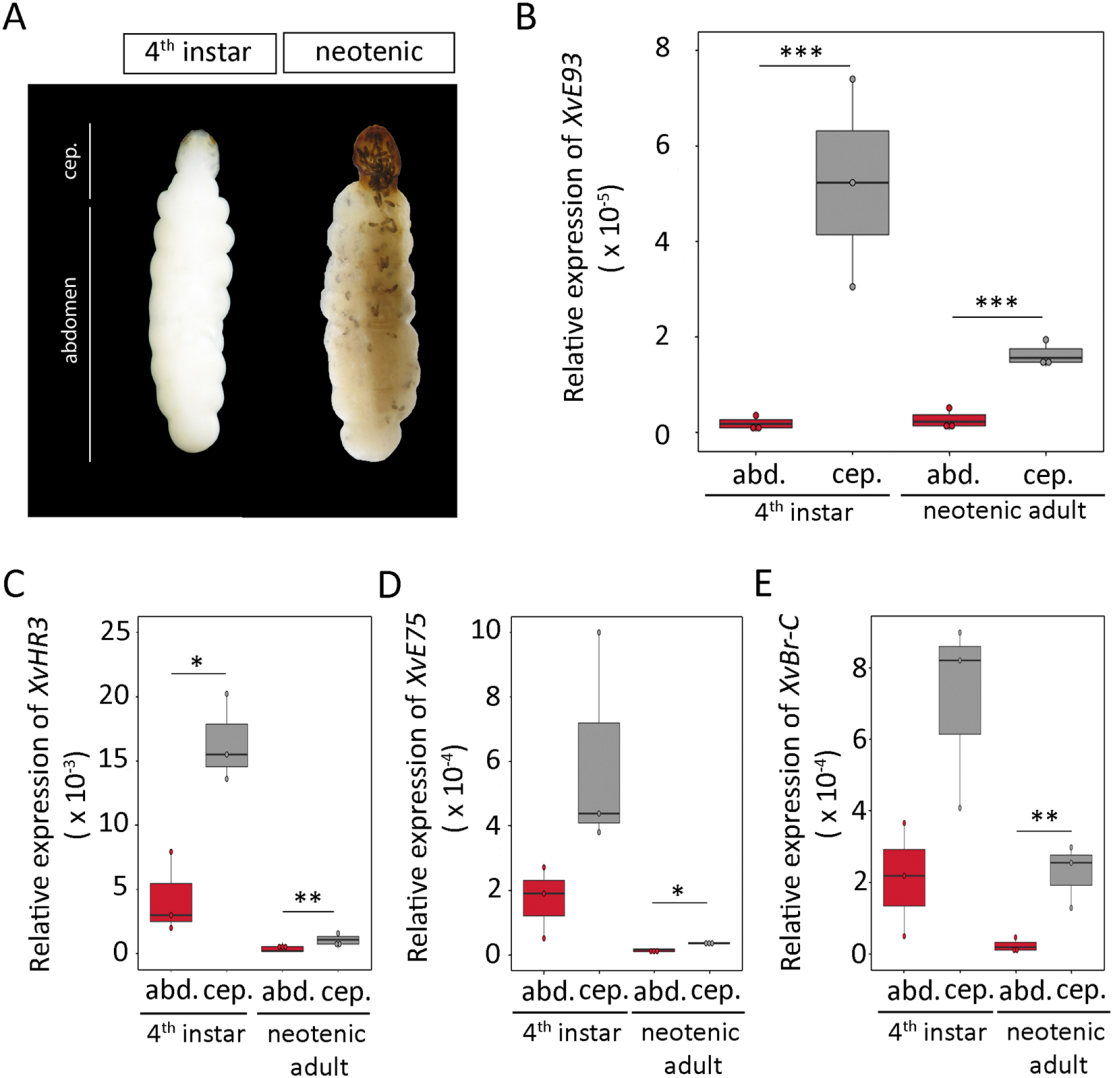
Expression levels of *XvE93*, *XvHR3*, *XvE75*, and *XvBr-C* in the cephalothorax and abdomen of *Xenos vesparum* females. (**A**) Dorsal views of a 4^th^ instar larva and a neotenic adult female. The abdomen (abd.) and the cephalothorax (cep.) regions are marked. (**B**–**E**) Transcript levels of (**B**) *XvE93*, (**C**) *XvHR3*, (**D**) *XvE75*, and (**E**) *XvBr-C* (without discriminating isoforms) were measured by qRT-PCR in the cephalothorax and abdomen of 4^th^ instar larvae and neotenic adult females. Transcript abundance values in panels B-E are normalized against the *XvRibosomal 18S* transcript. The range of expression is shown by Boxplot: boxes demarcate the upper and lower quartiles, while the heavy bar indicates the median value of normalized expression. Whiskers extend to the most extreme values. Points represent each individual measurement. Asterisks indicate differences statistically significant at *p ≤ 0.05; **p ≤ 0.01; and ***p ≤ 0.001 (*t*-test).

To characterize in more detail the expression pattern of *XvE93* in *X*. *vesparum* females, we next measured *XvE93* mRNA levels in staged 3^rd^, early and late 4^th^ instar larvae as well as in neotenic female adults. As expected, mRNA expression levels of *XvE93* were very low in 3^rd^ and at the beginning of the 4^th^ larval instars, but, surprisingly, the levels of *XvE93* were significantly up-regulated at the final part of the 4^th^ larval instar and the adult stage (Fig. 4B). This stage-specific up-regulation of *XvE93* is surprising in that, as previously stated, neotenic *X*. *vesparum* adult females are characterized for the lack of complete metamorphosis and the retention of an overall juvenile morphology. However, the female cephalothorax presents some adult-specific features (Fig. 5A), such as the pheromone glands and structures that support insemination and the release of the 1^st^ instar planidia. This observation raised the possibility that the late increase in *XvE93* levels would be related with the development of such structures. To test this hypothesis, we measured the transcript levels of *XvE93* separately in cephalothorax and abdomen of the late last juvenile instar, when *XvE93* was up-regulated (Fig. 4B), and the neotenic-adult stage. Remarkably, as shown in Fig. 5B, *XvE93* was significantly up-regulated in the cephalothorax of 4^th^ instar larvae and neotenic females but not in the abdominal part. This confirms the correlation between the specific increase in *XvE93* expression in the cephalothorax with the occurrence of adult-specific structures in this part of the neotenic female.

Next, we investigated how the expression of *XvE93* is differentially regulated in neotenic females. Since E93 expression is induced by 20E in holometabolous insects^35,48,52,53^, the differential expression could be explained by differences in the signaling, or response to 20E in different parts of *X*. *vesparum* female body. To test this, we analyzed the differential expression of *XvHR3* and *XvE75* (GenBank accession numbers MH220843 and MH220844, respectively), two 20E-dependent nuclear receptors of the stereotypic genetic cascade that responds to the 20E signal in insects^47,54^. Remarkably, the mRNA levels of both factors were higher in the cephalothorax of female 4^th^ instar larvae compared to the abdominal region (Fig. 5C,D). Higher levels of *XvHR3* and *XvE75* remained in the cephalothorax of neotenic females, although the levels of both factors were very low (Fig. 5C,D). Overall, these results suggest that higher levels of 20E signaling in the cephalothorax of last instar *X*. *vesparum* female larvae could be responsible for the particular increase in the expression of *XvE93* in this region, which, in turn, results in the “metamorphic” transformation of the anterior part of the body.

In all holometabolous insects studied to date, E93 is highly expressed specifically during the pupal and adult stages^1,34,35,48,53^. Functional studies have shown that E93 is required during the pupal period for proper adult differentiation, as RNA interference (RNAi)-mediated depletion of E93 prevented pupal-adult transition in *D*. *melanogaster* or even produced a supernumerary second pupa in *T*. *castaneum*^1,34^. Similar results were also observed in the lepidopteran *B*. *mori*^48^. Remarkably, the function of E93 is conserved in hemimetabolous insects^34,55^. In the hemimetabolous cockroach *B*. *germanica*, *BgE93* is highly expressed in metamorphic tissues during the last nymphal instar, and RNAi-mediated knockdown of *BgE93* in the nymphal stage prevented the nymphal–adult transition, inducing endless reiteration of nymphal stages^34^. Due to the evolutionary conservation of its expression and function, E93 is considered the “master factor” of adult metamorphosis in winged insects^34^. Importantly, the expression dynamic of *XvE93* in *X*. *vesparum* males reported in our work is consistent with the expression of *E93* in all holometabolous insects analyzed to date. In addition to the expression pattern of *XvE93* in males, it has been previously suggested that the expression of the pupal-specifier *XvBr-C* presents a consistent increase during 4^th^ larval instar and early pupa stages in *X*. *vesparum* males^36^, an expression pattern that is consistent with that observed in other holometabolous insects^28,29,32,56,57^. Altogether, these data show that male *X*. *vesparum* displays the typical holometabolous metamorphic development characterized by the sequential occurrence of Br-C during the larval-pupal transition and of E93 during the pupal-adult transition. In contrast, in females we observed very low levels of *XvE93* in the 4^th^ instar larvae and in neotenic females. Likewise, the expression of *XvBr-C* in females does not show a detectable induction in the same developmental period^36^. The absence in neotenic females of the typical holometabolous expression pulses of *XvE93* (this paper) and *XvBr-C*^36^ during the larval-to-adult transition correlates with the lack of the pupal stage, and clearly suggests that the suppression of the pupal determination program has contributed to the occurrence of female neoteny in *X*. *vesparum*.

It is interesting to note, however, that although *X*. *vesparum* neotenic females retain an overall larviform appearance at the posterior part of the body, the anterior extruded part of the body, the cephalothorax, undergoes a “metamorphic-like” transformation between the 4^th^ larval instar and the adult stages without an intermediate pupal stage. This transformation includes the development of structures to aid insemination, the release of the planidia as well as the pheromone glands^20^. Remarkably, we have found that the occurrence of such “metamorphic-like” process in the cephalothorax correlates with the significant increase of *XvE93*, *XvHR3* and *XvE75* expression in this part of the body (Fig. 5). Interestingly, we observed that *XvBr-C* was also up-regulated in the cephalothorax of 4^th^ instar larvae and neotenic females compared to the abdominal part (Fig. 5E), suggesting that a differential increase in 20E signaling in the cephalothorax results in higher expression levels of *XvE93* and *XvBr-C*, which controls the development of adult features. Altogether, our results show that the suppression of *XvE93* and *XvBr-C* expression in *X*. *vesparum* females, especially in the abdominal part of the body, may underlie the development of female neoteny in Strepsiptera.

A change in the expression of key developmental genes seems to be also underlying the evolution of progenesis, a second developmental strategy of paedogenesis that consists in the early growth and maturation of the ovaries during larval development. In the dipterans *Heterozepa pygmaea* and *Mycophila speyeri*, for example, a precocious up-regulation of the heterodimer EcR/USP in the ovaries of first instar larvae correlates with the early maturation of the ovary within the larval body in paedogeneic females^58^. Therefore, critical variations in the expression levels of genes that are important for the transduction of JH and 20E hormonal signaling may be responsible for the occurrence of paedogenesis in insects.

### Expression of *XvKr-h1* during metamorphic and neotenic development in *X*. *vesparum*

**Figure 6:**
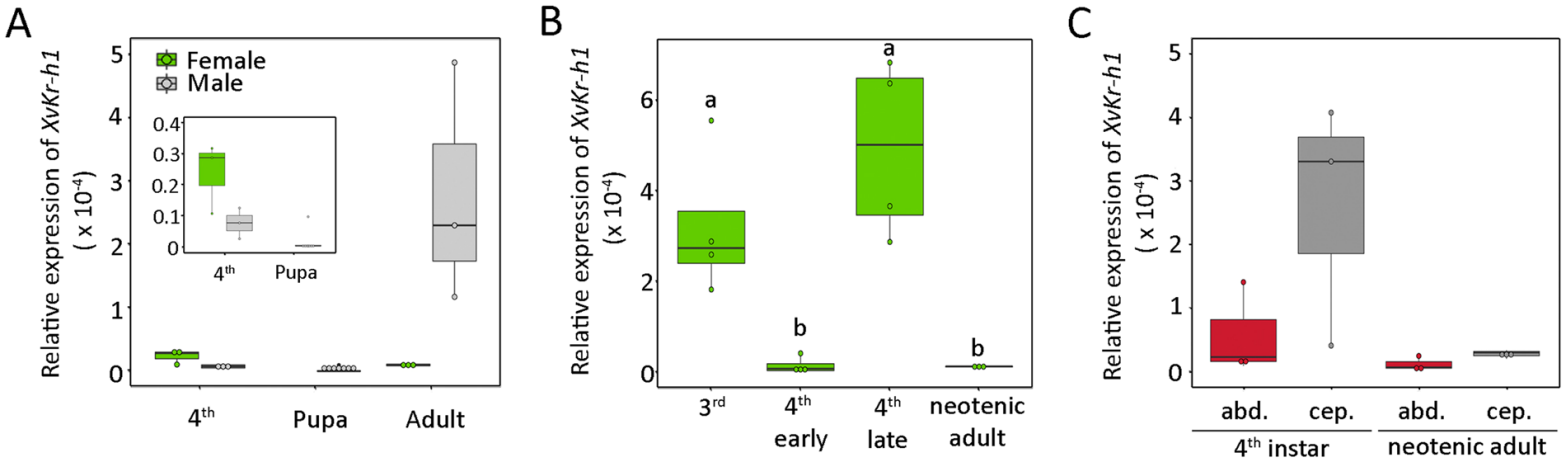
Developmental expression profiles of *Xenos vesparum XvKr-h1* in the larva to adult transition. (**A**) *XvKr-h1* mRNA levels were measured by qRT-PCR in the larva-to-adult transition in male (4^th^, pupa, adult) and female (4^th^ and adult) whole bodies. The inset panel shows a magnification of expression levels in 4^th^ larval instar and pupa. (**B**) *XvKr-h1* mRNA levels (qRT-PCR) in the whole body of 3^th^, early and late 4^th^ and neotenic females. (**C**) *XvKr-h1* mRNA levels (qRT-PCR) in the cephalothorax (cep.) and abdomen (abd.) of 4^th^ instar larvae and neotenic adult females. Transcript abundance values in all panels are normalized against the *XvRibosomal 18S* transcript. The range of expression is shown by Boxplot: boxes demarcate the upper and lower quartiles, while the heavy bar indicates the median value of normalized expression. Whiskers extend to the most extreme values. Points represent each individual measurement. Different letters in panel B represent groups with significant differences according to ANOVA test (Tukey, p ≤ 0.005). Asterisks in panel C indicate differences statistically significant at **p ≤ 0.01 (*t*-test).

Finally, to characterize the expression of the genes that form the MGN, we identified the transcription factor *XvKr-h1* in this species. To this aim, we isolated a fragment that encompasses seven zing-fingers of the DNA-binding domain through the assembling of *X*. *vesparum* transcriptomes (GenBank accession number MH220842) (Supplementary Fig. 2). We, then, analyzed the expression of *XvKr-h1* during the larval-adult transition in *X*. *vesparum*. In males, *XvKr-h1* mRNA levels were low in 4^th^ instar larvae, almost undetectable in the pupal period, and significantly high in the adult (Fig. 6A). In contrast, consistent with the absence of pupal stage in the neotenic female, *XvKr-h1* mRNA levels did not show differences between 4^th^ instar larva and the neotenic female (Fig. 6A). As before, we next analyzed in detail the expression levels of *XvKr-h1* in females and found that *XvKr-h1* was clearly detected in 3^rd^ instar larvae, but its levels significantly dropped at the onset of the 4^th^ larval instar, only to reappear at the final part of the instar (Fig. 6B). In the neotenic adult female, low levels of *XvKr-h1* were detected (Fig. 6B). Interestingly, although not significant, the increase in *XvKr-h1* expression in late 4^th^ larval instar was higher in the cephalothorax compared to the abdominal region, while in neotenic animals very low levels of *XvKr-h1* were detected in both parts of the body (Fig. 6C).

Similar to XvE93, the expression pattern of *XvKr-h1* in the male is comparable to those observed in holometabolous insects, in which *Kr-h1* is expressed through larval development to become strongly repressed by E93 during the pupal stage, and to reappear during the adult stage^1,26,27,59^. On the other hand, the levels of *XvKr-h1* in females display a particular dynamic: at the early last larval stage, the decline of *XvKr-h1* is similar to that observed at the onset of the last larval instar of holometabolous insects; however, at the final part of the 4^th^ instar, *XvKr-h1* is up-regulated in the cephalothorax region in parallel to the increase in the expression of *XvE93* and *XvBr-C*. This result suggests that in the neotenic female, XvKr-h1 has lost the strong repressive activity upon *XvE93* and *XvBr-C* expression that is observed in holometabolous insects studied^1,35^. Alternatively, it is plausible that the up-regulation of *XvKr-h1* in 4^th^ instar larva is necessary to prevent a stronger transcriptional induction of *XvE93* that would result in the activation of the adult genetic program in the body of female *X*. *vesparum*.

## Concluding Remarks

Loss of complete metamorphosis in the strepsipteran suborder Stylopidia has evolved exclusively in females. Males, however, display the typical holometabolous larva-pupa-adult transition. Correlating with the presence of the metamorphic pupal stage in *X*. *vesparum* males, high levels of expression of the adult specifier *XvE93*, along with the disappearance of the anti-metamorphic *XvKr-h1*, are detected in the pupal stage. In contrast, very low levels of *XvE93* are observed throughout development of *X*. *vesparum* females. However, a specific up-regulation of *XvE93* and *XvBr-C* in the cephalothorax of late female 4^th^ instar larva correlates with the occurrence of adult-specific features in the anterior part of the neotenic female body. Overall, our results, together with previous work^36^, suggest that neoteny in strepsipteran females arose by the suppression of the genetic program that controls the formation of the pupa, that is the sequential expression of *XvBr-C* and *XvE93* factors at the end of larval development. The loss of expression of *XvE93* and *XvBr-C* in *X*. *vesparum* females could be the result of changes in the endocrine milleu, with alterations in the titer of JH and/or 20E in particular stages of development. There might also be changes in the regulation of the expression of these genes, as suggest by the fact that XvKr-h1 does not seem to act as a potent repressor of *XvE93* and *XvBr-C* in females. In summary, our work highlights the importance of the MGN in the regulation of insect metamorphosis and in the evolution of insect neoteny in the order Strepsiptera.

## Electronic supplementary material

Supplementary Information
