# Supplementary material for: Differential expression of the adult specifier E93 in the strepsipteran Xenos vesparum Rossi suggests a role in female neoteny
Source: Sci Rep. 2018 Sep 21;8:14176. doi: 10.1038/s41598-018-32611-y (PMC6155025; doi:10.1038/s41598-018-32611-y)
Supplement: Supplementary file 1 — Supplementary Information [file 41598_2018_32611_MOESM1_ESM.pdf]

SUPPLEMENTARY INFORMATION

Differential expression of the adult specifier E93 in the strepsipteran *Xenos vesparum* Rossi suggests a role in female neoteny

Chafino S., López- Escardó, D., Benelli, G., Kovac, H., Casacuberta, E., Franch-Marro, X., Kathirithamby, J*. and Martín, D*.

Supplementary Table 1. Accession numbers of E93 sequences used for alignments.

Taxa	Order	Accession number
Ixodes scapularis	Acari	XP_002435807.1 (IscW_ISCW019047)
Parasteatoda tepidariorum	Araneae	XP_015926289.1
Blattella germanica	Blattodea	CCM97102.1
Aethina tumida	Coleoptera	XP_019872192.1
Agrilus planipennis	Coleoptera	XP_018321151.1
Dendroctonus ponderosae	Coleoptera	XP_019771885.1
Leptinotarsa decemlineata	Coleoptera	AQN67828.1
Nicrophorus vespilloides	Coleoptera	XP_017770390.1
Tribolium castaneum	Coleoptera	KYB25179.1
Aedes aegypti	Díptera	XP_001649373.1
Culex quinquefasciatus	Díptera	XP_001863525.1
Drosophila melanogaster	Díptera	NP_652002.2
Drosophila simulans	Díptera	EDX13858.1
Acromyrmex echinatior	Hymenoptera	EGI58724.1
Apis mellifera	Hymenoptera	BAB64310.1
Bemisia tabaci	Hymenoptera	XP_018900968.1
Bombus impatiens	Hymenoptera	XP_003490553.1
Cyphomyrmex costatus	Hymenoptera	KYN00623.1
Eufriesea mexicana	Hymenoptera	XP_017758118.1
Trachymyrmex zeteki	Hymenoptera	KYQ50221.1
Zootermopsis nevadensis	Isoptera	KDR22086.1
Amyelois transitella	Lepidopter	XP_013188059.1
Bombyx mori	Lepidopter	AIL29268.1
Operophtera brumata	Lepidopter	KOB75508.1
Papilio machaon	Lepidopter	KPJ20458.1
Xenos vesparum	**Strepsiptera**	**MH220841**

Supplementary Figures

1	MAECSFTRCIQQRRFIRKQLRKWTTNMVHIVGLERLAEELMGQKKWQLYRDSMANNQEICN	60
61	NNLPNISDKHNNNIDGNEHGDHDREQDIHDWSPNTPCQFCADGVLGHDHNGTFMSPQAS	119
120	DCDSSDSDLDLAPTLLNPASLYESKNINNLFGKHFSSNNLNSMATSLAAVSALNNSTSLFPNNW	183
184	YFSQFPKHFPLSHSNSLSTSDSVHEMNRHFIEQ**PLDLSAK**NSLNSQSPDSQQPLQQAQLQQP	245
246	SPQLQQAPQQHTSQIQKLINPGARLSTPNFDNRHIFKAKPRMSSVAGRRTYTEDELQAALRDI	307
308	QSGKLGTRRAAVIYGIPRSTLRNKVYKLAMEREKETLLNNQNAIVDLKPTLKIEDDDEMIDEDK	371
372	DLSGAEDEKEVEKALQQPLITIADILRFSNLNNEDN**LRNLL**HKCHESQEFLTSINFEHNPLGPF**L**	436
437	**QSLL**LSGKFQQNPHHSEQQLNNDDLQSKTSSSPTPQSVIVKNENVISKPQDVNEKDEESCMS	499
500	NVILKIPSFRPSSTSSSSLSSIAAVQPNIKPPSLNLNVSCNNEIASTSFRNSAESSMVLTSPITSDSS	567
568	PIPSIMNSATAITPTVTPIQTATPSLPNATNKNISLRDVIARSINQNYPQSSATANESLKTIEMDFK	633
634	RQFAAPPLAILNNHNHSDRNFFGKGFSPSNSNMNNPNNSQGG**KGTRPKRGKYRNYDRDSL**	693
694	**VEAVRAVQRGEMSVHRAGSYYGVPHSTLEYKVKERHLMRPRK**RDPKPSTTNLDEKSTSSTM	755
756	ASNKNIPVDNKNKQQITPNLLNKPQAVQKFSPTTSPNGIKMFEPTAAPMNYVPPQFPFWHPS	816
817	FHNLSMDYGRSSSNHPHSHTHPPPHPPPPPPPSFPLNTEQFFNAQMLHKLQQQQQQEPDV	876
877	QIKAVEEPSILDGIIRSSLETNQKQYQTQNMDTTENKVLLEQLCRNSNRLTPQPTNSTNNYLQE	940
941	ANSSSNDSFQSSKRNVSPKHSPQTDNPFLVVSNNNNNNNNNNNNNERVIVKDEIESDESTNH	1001
1002	VPLNNEQTDKADGDAERLQPEESVKMLAMSSNTGSNNGNGSSEFFANFKKDNNAMFMEK	1060
1061	NGLFETAKVCASESNFDLVQD	1083

Supplementary Figure 1. Amino acid sequence of *Xenos vesparum* XvE93. The two HTH-DNA binding motifs, RHF1 and RHF2, are underlined, and RHF2 is in bold. The two NR-boxes are marked in red. The CtBP-interaction motif is marked in green.

1 NNSNEDIYQ**CNMCLKTFTVLARLTRHYRTH**TGEKPFQ**CEFCSKSFSVKENLSVHRRIH**TK 60
61 ERPYK**CEICSRAFEHSGKLHRHMRIH**TGERPHK**CTICEKTFIQSGQLVIHMRTH**TGEKPY 120
121 V**CTICKKGFTCSKQLKVHSRTH**TGEKPYS**CDICGKSFGYNHVLKLHQVAH**YGEKVYK**CTI** 180
179 **CKDTFTSKKSMEIHIKNH**SDSTSHTNHQSSTTASSEIQQSEAHVEKHEGLRYVLQQPKQ 239
238 SDKHLYNLLTHPHEFKQDLATIAHKAPVHVSVPAVHDHVEDKISNSNQQHQPLTPPSSNS 298
299 SSPAGSPAMQQTVDLETDDDQHN**LPLRKR**SKMFLKSFETSTVSVPSIPPNNVNYPQIP 357
358 IRYNTVIHYAKAS 370

Supplementary figure 2. Partial amino acid sequence of *Xenos vesparum* XvKr-h1. Seven C2H2-zinc fingers domains are underlined and the A-motif in the N-terminal region, which is typical of Kr-h1 proteins, is marked in red.
